# MANTA—an open-source, high density electrophysiology recording suite for MATLAB

**DOI:** 10.3389/fncir.2013.00069

**Published:** 2013-05-06

**Authors:** B. Englitz, S. V. David, M. D. Sorenson, S. A. Shamma

**Affiliations:** ^1^Neural Systems Laboratory, Institute for Systems Research, University of MarylandCollege Park, MD, USA; ^2^Equipe Audition, Département d'études cognitives, Ecole Normale SuperieureParis, France; ^3^Laboratory of Brain, Hearing and Behavior, Oregon Hearing Research Center, Oregon Health and Science UniversityPortland, OR, USA; ^4^Department of Electrical and Computer Engineering, University of UtahSalt Lake City, UT, USA; ^5^Blackrock MicrosystemsSalt Lake City, UT, USA

**Keywords:** data acquisition, neural recordings, electrode arrays, LFP, μECoG

## Abstract

The distributed nature of nervous systems makes it necessary to record from a large number of sites in order to decipher the neural code, whether single cell, local field potential (LFP), micro-electrocorticograms (μECoG), electroencephalographic (EEG), magnetoencephalographic (MEG) or *in vitro* micro-electrode array (MEA) data are considered. High channel-count recordings also optimize the yield of a preparation and the efficiency of time invested by the researcher. Currently, data acquisition (DAQ) systems with high channel counts (>100) can be purchased from a limited number of companies at considerable prices. These systems are typically closed-source and thus prohibit custom extensions or improvements by end users. We have developed MANTA, an open-source MATLAB-based DAQ system, as an alternative to existing options. MANTA combines high channel counts (up to 1440 channels/PC), usage of analog or digital headstages, low per channel cost (<$90/channel), feature-rich display and filtering, a user-friendly interface, and a modular design permitting easy addition of new features. MANTA is licensed under the GPL and free of charge. The system has been tested by daily use in multiple setups for >1 year, recording reliably from 128 channels. It offers a growing list of features, including integrated spike sorting, PSTH and CSD display and fully customizable electrode array geometry (including 3D arrays), some of which are not available in commercial systems. MANTA runs on a typical PC and communicates via TCP/IP and can thus be easily integrated with existing stimulus generation/control systems in a lab at a fraction of the cost of commercial systems. With modern neuroscience developing rapidly, MANTA provides a flexible platform that can be rapidly adapted to the needs of new analyses and questions. Being open-source, the development of MANTA can outpace commercial solutions in functionality, while maintaining a low price-point.

## Introduction

One and a half centuries after Caton (1875) measured the first extracellular potentials, the electrical readout of brain activity is still one of the primary experimental approaches used in neuroscience, offering high temporal precision and a range of spatial granularity spanning single unit recordings, local field potentials (LFP), micro-electrocorticograms (μECoG), electroencephalography (EEG) and *in vitro* micro-electrode array (MEA) recordings. Each of these techniques' spatial coverage/resolution can be optimized by recording simultaneously from hundreds of sites using state-of-the-art probes, such as micro-electrode arrays, high-density μECoG surface arrays or many-channel EEG caps. The availability of large numbers of channels is not only important for deciphering neural codes, but also enables single trials decoding approaches for future brain computer interfaces (Nicolelis and Lebedev, 2009; Mesgarani and Chang, 2012).

As modern probes integrate many channels (Du et al., 2011), the number of sites keeps increasing rapidly (Buzsaki, 2004; Stevenson and Körding, 2011) and researchers are faced with the challenge to choose an appropriate system to record and visualize the data. Several companies offer out-of-the-box systems designed to handle the substantial amounts of data generated from these recordings. However, these systems are usually closed-source and offered at high prices, especially when hundreds of channels are to be recorded. An open-source solution brings many advantages with it, foremost the possibility to efficiently adapt the system on a local basis, collaborative improvements, and substantial reductions in costs. Many software projects—such as Linux, OpenOffice, Apache and Firefox (e.g., Raymond, 1999)—have demonstrated the surprising effectiveness of community based code-development. For an open-source solution to be successful in the domain of neurophysiology, the question becomes whether general purpose hardware is capable of processing and displaying the large amounts of data in real-time.

For an open-source system to be practical, suitable hardware for computation/graphics and data acquisition (DAQ) must be available. On the computation and graphics side, the super-computing and gaming markets have made high end CPUs and GPUs available at quite low prices. On the DAQ side, multiple companies—most notably National Instruments—offer a wide range of highly efficient, easily accessible DAQ cards at reasonable costs, which can be bundled to support recordings from hundreds to thousands of channels (at the sampling rates of interest).

On this basis we have developed an open-source DAQ system, which is easy to extend and use. MANTA is written in MATLAB and makes use of a range of efficient coding strategies to allow fast plotting for 100's of channels. It supports natural visualization of arbitrary 1D, 2D and 3D electrode array geometries, integrates filtering, spike-sorting and denoising for online display, synchronization and communication with a control unit, and can acquire data from both analog and digital headstages. It is built using a modular design to facilitate and encourage the addition of new hardware and analysis tools. Being based on MATLAB, debugging and improving MANTA is a rapid process aided by an integrated simulation mode, and is thus easily accessible to the plethora of neuroscientists versed in MATLAB. MANTA is licensed under the GPL and can be freely used and improved (see code.google.com/p/manta-system).

Here, we detail its structure, operation and features, quantify its cost, performance and reliability, and demonstrate its capabilities using some recordings performed with it.

## Methods

### System configuration

**Figure 1:**
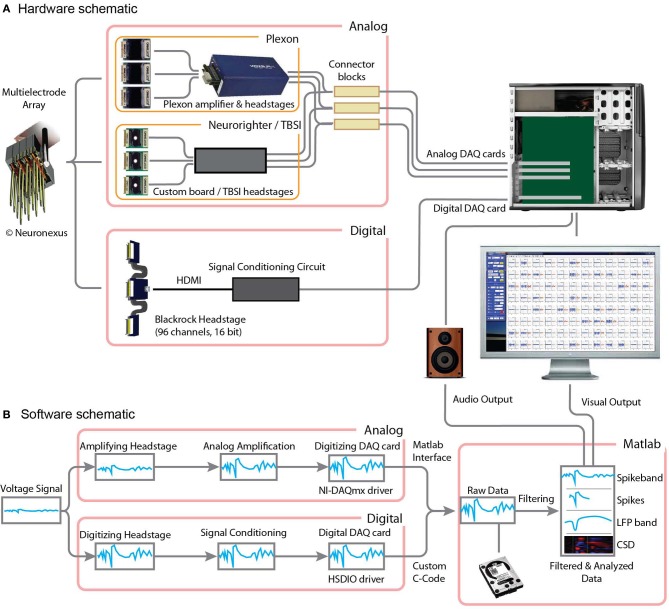
**Digital and analog recording configurations compatible with MANTA. (A)** A high channel-count electrode, such as a 3D probe by NeuroNexus or a Utah array by Blackrock is connected to an analog or digital headstage. For analog headstages (top) the signal is further amplified using a second amplifier stage (e.g., Plexon or Neurorighter are shown here). The output of these systems is then routed into National Instruments DAQ cards (M- or X-series). For the digital headstage (bottom), the signal is conditioned using a special circuit (see Figure A2) and then digitized using a high-speed digital DAQ card by National Instruments (656X series). The signals are treated uniformly in MANTA and can then be displayed or sent to audio output. **(B)** The data flow from the probe to the display is represented schematically here. In the analog path (top), the signal is amplified, but remains analog until it reaches the DAQ card. In the digital path (bottom), the signal is directly digitized at the headstage and then relayed digitally to the digital DAQ card. Next, the raw data is read into MANTA (using custom code to interact with the NI drivers) and directly streamed to disk, followed by several filtering and analysis steps before the data is displayed. The last steps require the MATLAB Signal Processing Toolbox.

Assembling a MANTA system requires making a few choices, including the workstation configuration, headstage type (digital/analog, and for analog headstages the second level amplifiers) and vendor, and choosing an appropriate DAQ card, schematically shown in Figure 1 (see more details below).

MANTA can be used with either analog or digital front-end amplifiers (headstages) and runs on 32-bit and 64-bit platforms. Windows is fully supported. On Linux and Mac OS X, National Instruments only provides limited support for the analog M-series cards (NI-DAQmx base driver), hence only the analog section below applies to these operating systems. In addition to MATLAB itself, the Signal Processing, the Instrument Control (for TCP/IP communication) and the DAQ (for audio output) toolboxes are required, although we are working to overcome this dependence. A detailed comparison of the three systems outlined in Figure 1 is given in Table 1.

**Table 1:** **Overview of system configurations**.

**System**	**Headstage + Cable**	**Preamp**	**DAQ card + Cable**	**Cost/Channel (asymptotic)**	**System cost (192 Channels)**	**Max. sample rate (Hz)**	**Noise level**	**Max. channels**
Analog (Neurorighter)	$1700/32Ch	$100/32Ch	$1800/32Ch	~90	~20000	25000	6.3 μV	224 (7× PCI-6255)^*^ 576 (18× PXI-6363)
Analog (Plexon, BR)	$2000/32Ch	$2500/32Ch	$1800/32Ch	~180	~35000	25000	2.1 μV	224 (7× PCI-6255)^*^ 576 (8× PXI-6363)
Digital (Blackrock)	$7000/96Ch	$50/96Ch	$3600/1536Ch	~85	~20000	31250	3.0 μV	1536 (1× PCI-6561)

* Using only internal PCI/PCIe slots.

All results reported below were collected on a PC workstation (Core i7 2700 K, GeForce GTS 460, current cost <$1000) running Windows 7 32-bit/64-bit, MATLAB 2010b and the National Instruments drivers NI-DAQmx 9.5/HSDIO 1.8.1. If newer hardware were used, the performance would improve accordingly. Prices for other hardware cited below are accurate as of 02/2013 and are likely to decrease as commercial DAQ hardware continues to improve.

#### Analog headstage

Since the introduction of VLSI headstages, a range of companies (TBSI, Blackrock, Tucker Davis Technologies, Plexon, Multichannel Systems) have started offering analog headstages in various configurations, regarding the number of channels (typically 16 or 32), amplification (ranging from 1× to 100×), and connector type [mostly Omnetics Nanostrip and ZIF (zero insertion force) clip]. All of these are in principle compatible with MANTA, as long as the channels are sufficiently amplified and can be individually connected to the break-out boxes (NI SCB-68 or a similar screw terminal DAQ interface). While analog VLSI headstages typically have excellent noise characteristics, the requirement of individual lines per channel in the headstage cable becomes increasingly problematic, the more channels are used. Pricewise, analog headstages are at this point typically less expensive than their digital counterparts (although this is likely to change within the next few years). Analog headstages bring the advantage of simpler, potentially more robust operation, due to the relatively straightforward characteristics of the amplification circuitry and the direct readout on the DAQ card side.

After the headstage a second stage of amplification/filtering is often required. MANTA has been tested with both Plexon amplifiers and the boards designed for the Neurorighter system (Rolston et al., 2009), which provides open-source, analog amplifier and stimulator boards (see Discussion for more details). Both second-stage amplifiers provided similar noise levels. While the Plexon system provided a few more referencing options, the Neurorighter system can easily be extended to stimulate electrically as well.

A range of analog DAQ cards can be used for the various applications, and the best choice depends on the required sampling rates and number of channels. For high sampling rate applications (single cell, MEA, 20 and 30 kHz) various National Instruments M-series (e.g., PCI/PXI 6254, PXI/PCI/e 6259) and X-series (e.g., PCI/PXI 6363) cards can be used, which each offer 32 channels per card at a maximum of 31.25 kHz/channel (~$35/channel). For lower sampling rate applications [LFP, μECoG, magnetoencephalographic (MEG), EEG] the PXI/PCI 6255 cards can be used, which can process 80 channels at 9.375 kHz (at only $25/channel).

The maximal number of channels is limited mostly by the number of available PCI/PCIe or PXI (if an external PXI chassis is used, which is available for all operating systems) slots. For PCI/PCIe slots, the form-factor of current mainboards allows up to ~8 internal slots, one of which will hold a graphics card, thus leaving 7 slots for DAQ cards, equivalent to 224 = 7 × 32 channels. For PXI slots, the number of channels can be very high, since multiple PXI chassis can be connected. Assuming only a single chassis 576 = 18 × 32 channels can be utilized (NI PXI-1045).

#### Digital headstage

Recently, a few companies (Blackrock, Tucker Davis Technologies) have started offering digital headstages, which have some advantages over analog headstages. Firstly, no noise is added on the way to the DAQ system, since the transmitted signal is already digitized. This is particularly important for experiments with freely moving animals. Secondly, the digital signals are multiplexed on a single or a few lines, which keeps the total number of wires in the headstage cable much lower than the number of channels. Again this is advantageous under conditions where a light and flexible cable is beneficial. Thirdly, since the digitization is already performed at the headstage, no additional amplification stage is needed. This simplifies the system setup and reduces its costs.

**Figure 2:**
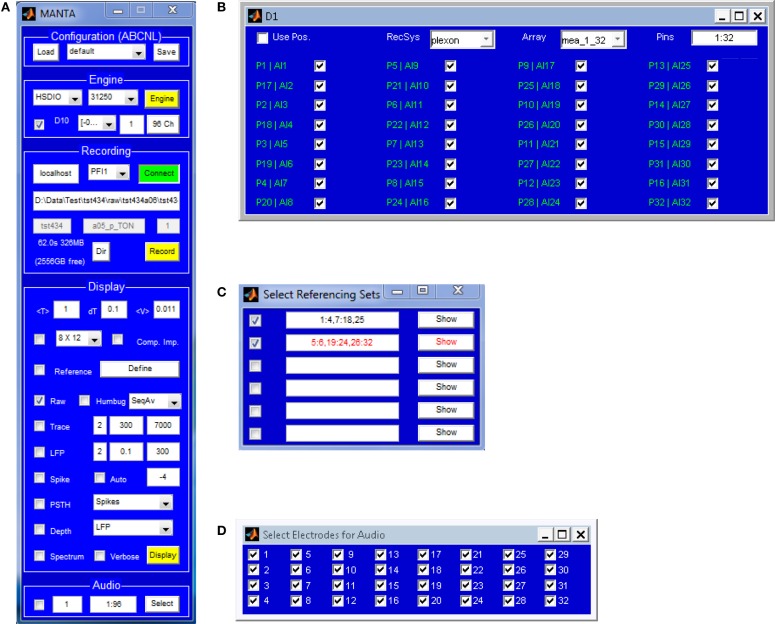
**The main window **(A)** of MANTA consists of 5 panels (lighter blue areas): configuration, Engine, Recording Display and Audio.** In the Configuration panel, system configurations can be saved for later use and loaded again. Essentially all settings in MANTA can be controlled from the GUI. The Engine panel controls which data acquisition hardware is used, selects channels **(B)**, sets parameters of the acquisition such as sampling rate or input range, selects electrode array layout, and starts and stops acquisition. The Recording panel serves a dual purpose: to allow manual recordings and display information about the current recording when controlled remotely. The Display panel holds the options for the graphical representation of the data, which can be modified/toggled during an active acquisition session, without interrupting acquisition. Options include to filter the signal within different bands (typically LFP and spike band), trigger spikes, common mode notch filtering, Fourier spectrum display, and common average referencing for several sets of channels **(C)**. The Audio panel allows to select a subset of channels and output to computer speakers. An audio selection panel can be opened to individually select the channels to play (**D**).

Presently, MANTA only supports the digital protocol used in Blackrock headstages. To relay the digital signal to and from the headstage with minimal distortion it is necessary to interject a custom built circuit for signal conditioning/separation between the DAQ card and the headstage. The circuit diagram is provided as Figure A2 and the PCB-board definitions (in the format used by expresspcb.com) can be found in the software repository (InterfaceBoardDesignX.Y.pcb). The costs for making this box are ~$60 and its assembly only requires a reasonable acquaintance with soldering (two chips have to be SMD soldered). While the lack of additional amplification reduces costs, digital headstages are still more expensive per channel than their analog counterparts (roughly by a factor of 2). Overall, the digital system is on par in terms of costs with the least expensive analog system (Figure 2, Blackrock vs. Neurorighter).

The maximal number of channels is comparably high in this case, since each digital DAQ card (e.g., a NI PCI-6561) can handle 1440 = 15 × 96 channels (via 15 digital lines, the 16th being reserved for digital triggering) and multiple cards could be used per computer. These numbers are almost a factor of 10 above commercially available arrays, although they require just one DAQ card.

### Software architecture

The complexity of a software package determines the likelihood for new developers to participate in an open-source project. To this end, MANTA was designed with simplicity and flexibility in mind. MANTA takes advantage of MATLAB's extensive library of mathematical transformations so that the system currently encompasses <5000 lines of code (including comments/empty lines etc.). New developers can thus learn the software in a short time before adding their own custom features.

**Figure A1:**
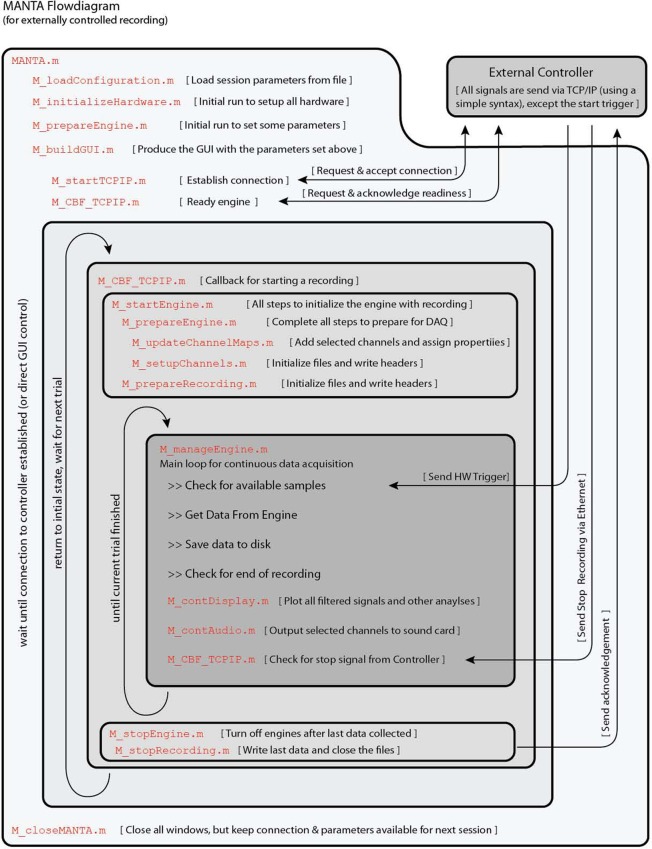
**Flow diagram of MANTA**.

**Figure A2:**
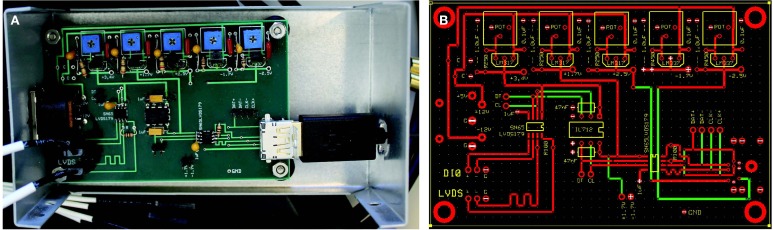
**Circuit diagram of the interface board for the Blackrock digital headstages. (A)** The finished intermediate box with the digital input and output channels entering at the left and the HDMI cable connecting to the headstage on the right. The potentiometers at the top only need to be set once to the correct voltages. **(B)** Circuit diagram of the circuit on the left from the expresspcb.com software. After submission to the company, the finished circuit boards arrive a few days later at an approximated cost of $30 per board. The components add ~$30, including the box.

The main DAQ loops are written to dynamically adapt their speed to the current load, determined by the number of channels, the chosen display options, and computer performance. All graphics and audio output are embedded inside try-catch environments, which shield acquisition from potential display errors. The flow of a recording session, including the main acquisition loop, is shown in Figure A1, which also includes the main functions called at each point in the sequence. The diagram serves as an introduction for programmers to important functions and the general flow of MANTA's execution.

Extensibility is eased by the use of function handles, e.g., exchanging the spike-sorter merely requires exchanging the currently used function handle by the custom written function with a matched interface.

All state information is collected in a global variable, designed to allow straightforward interaction with the GUI, improve performance and provide immediate access for all functions to the state of acquisition and the GUI. This choice also allows for compact callback functions, and provides an effective protection against hard-coding, since all parameter values are globally available. Name space pollution is avoided by collecting everything into one large data structure.

### Noise levels

The noise level determines the fidelity with which neural responses can be distinguished from background noise. The noise level in the present system is largely governed by the noise of the headstages themselves, since the signal arrives amplified/digitized at the DAQ cards. Both analog and digital headstages have noise levels of a few microvolts (μV, input referred, i.e., correcting for amplification), e.g., the TBSI 100× headstage is rated at 6.2 μV, the Blackrock analog headstage at 2.1 μV, and for the Blackrock digital headstage 3.0 μV. Overall, the noise level should be comparable if not identical to commercial systems, given that the same headstages and similar DAQ cards are used in most systems. The noise level after band-pass filtering the spike-band (300–7000 Hz) has an approximate noise level of 7–10 μV in the brain, which includes the contributions of neural background activity.

### Physiological recordings

We have recorded from many preparations using multielectrode arrays. Presently, we describe a high density recording from an awake ferret. A headpost implant and a craniotomy were performed on a ferret (as part of another study) as described previously (Fritz et al., 2003). A 96 (16 × 6) channel microelectronic array (Microprobes Inc., 2.5 MOhm, ø = 125 um) was advanced into primary auditory cortex A1 (thalamorecipient layers, ~600 um) while the ferret was passively listening to several stimulus paradigms (random tone sequences, TORC stimuli). Detailed spike sorting was performed *post-hoc*, yielding 56 single units.

## System properties and results

In the following sections we detail the user-level features of MANTA, the interface, reliability, a small user survey, system costs, setup time, and provide examples of real and simulated (for development) recordings.

### Features

The following list provides an overview of the features currently implemented in MANTA to illustrate the flexibility with which it can be adapted to the requirements of a certain type of recording, here, for single unit extracellular/LFP recordings. While many will be useful for μECoG and EEG recordings as well, some additional features or analyses should be added in these cases (e.g., head based representations and predefined referencing to certain electrodes). Most features below can be dynamically en-/disabled during recording.

- *Many channels:* A large number of channels (576 for analog and 1440 for digital headstages) can be acquired at sampling rates sufficiently high (>25 kHz) for identification of neural action potentials. The channel display count is limited primarily by monitor space rather than low level data processing.
- *Protected acquisition:* Raw data are saved to disk with high priority. Both video and audio outputs are enclosed by try-catch statements, such that potential errors during debugging or development do not affect acquisition.
- *User-friendly:* Almost all features are accessible via a graphical user interface (GUI) and thus can be controlled without programming experience. All GUI items have mouse-over help to provide rapid information while minimizing the use of valuable screen space by static labels.
- *Raw data display:* The raw voltage trace can be displayed, which allows early stage debugging of noise sources in the system.
- *LFP display:* A bandpass filtered voltage trace can be displayed (with variable filter properties), typically used to display the LFP, i.e., the signal low pass filtered at 300 Hz.
- *Spikeband display:* Another bandpass filter voltage can be displayed (again with variable filter properties), typically used to display the spike band, i.e., the signal band pass filtered between 300 and 6000 Hz.
- *Spike display:* Spikes can be triggered by an automatic or manual threshold (per channel basis) and displayed adjacent to each channel trace, along with an instantaneous measure of spike rate. Spike times are automatically written to disk for fast online analysis outside of MANTA.
- *Spike-sorting:* Spikes are sorted online using a fast, heuristic algorithm, which takes a limited number of properties (amplitude of maximum and minimum, zero-crossing) of each triggered waveform event into account, combines those in a one dimensional variable and clusters in this dimension up to four different spikes per electrode. User intervention is not required, since with >100 channels users are unlikely to spike-sort individual channels during recording. The interface for the online spike sorting is general, such that other sorters can be quickly implemented by supplying a function handle to the new sorter.
- *PSTH display:* The Post-Stimulus Time Histogram over multiple trials is automatically collected and can be overlaid on the voltage trace for each channel. Either spike events or the LFP signal can be averaged here. At this point different trial conditions are not distinguished, but can be added, if the conditions are transferred from the controller or can be decoded from the filename.
- *CSD display:* Current source density (CSD, Nicholson and Freeman, 1975; Pettersen et al., 2006) is a valuable tool for estimating the relative location of a multichannel probe in the cortex. Based on the array geometry, electrodes which are at different depth but in the same planar location are automatically combined to compute a CSD per shaft. Alternatively, the LFP can be displayed as a function of depth in a heat map.
- *Spectrum display:* The short-term power spectrum of the raw data can be plotted underneath each channel to visualize the instantaneous frequency content of the signal, thus providing information on the often studied bands (alpha, beta, gamma, delta, and theta) on a channel by channel basis.
- *Channel zoom:* Individual channels can be transferred to a new large window dynamically. This allows closer inspection of individual channels, which becomes more important if 100+ channels are recorded and the individual plots become small. If the single channel plot window is closed, the channel returns to the main plot window automatically.
- *Digital line noise filter (humbug):* While line noise is usually not much of an issue with VLSI headstages, a narrow band, digital filter can be enabled which effectively removes line noise (50 or 60 Hz). Multiple filters are available, and new filters can easily be implemented. Another, consecutive averaging filter can be enabled to deal with non-sinusoidal, but regularly repeating noise.
- *Common average referencing:* Since some noise sources feed into all channels, the common average across all channels (or arbitrary subsets from arbitrary subsets) can be subtracted. Together with the digital line noise filter, this subtraction can compensate substantially for noise present due to grounding issues or common input.
- *Audio output:* An arbitrary subset of channels can be selected and routed to the default audio output of the computer. MANTA uses a peak selecting algorithm, which avoids spikes being averaged out when multiple channels are audible.
- *Array geometries:* Multi-electrode arrays come in many shapes and configurations. MANTA offers a syntax to specify arrays in 1D, 2D, and 3D. The array geometry is then used to arrange the plots on the screen. For 3D arrays (e.g., for electrodes in an EEG cap and 3D multi-electrode arrays), the array can be rotated in 3D. The geometric arrangement makes it easy to associate properties of recording sites with the larger geometry of the brain, and also allows to account for the orientation at which the array is inserted. Further, the mapping of electrodes to pins (which is not always the same for different manufacturers) can be specified when defining the array, and thus decouple the implementation of the array from its geometry.
- *Communication with controller:* A TCP/IP connection and protocol is provided for connection with stimulus control programs. The protocol permits the control of MANTA's acquisition from another program, including the transfer of variables from MANTA to the controller. A set of functions to control MANTA is provided (see following section *Controlling Acquisition* for details).
- *Saved configurations:* Configurations of MANTA can be saved and loaded easily and thus allows the use of multiple configurations on a single experimental system, or by multiple users with different preferences.
- *Simulation mode:* Surrogate neural data can be generated online including slow oscillations, line noise, background noise, and realistic spikes from multiple units for testing on all computer architectures.
- *Extensibility:* MANTA makes extensive use of separate functions and callbacks, which simplifies the modification of functionality that may be called at multiple points in the processing cycle. As much as efficiency considerations permit, Interactions with the stimulator and DAQ calls are abstracted, such that they can be replaced as long as the interface is kept the same.

### Interface

**Figure 3:**
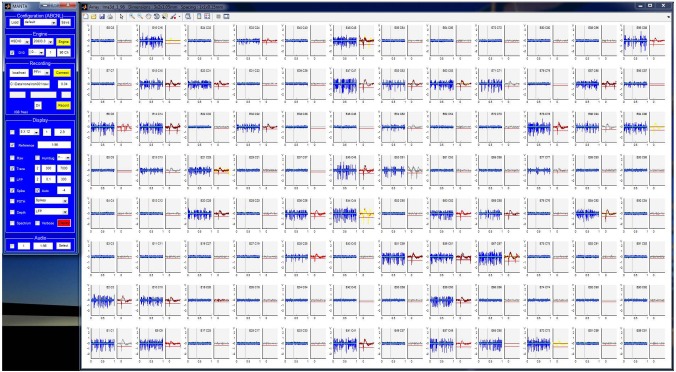
**The display window of MANTA is an array of plots, each corresponding to a single recording channel.** The layout of the plots follows the geometry of the electrode array and thus provides a sense of the spatial relation of the recorded signals. Plots split and resize appropriately if spike triggering or spectrum display is activated. The y-scale of all plots can be magnified together (mouse wheel) or individually (clicking below/above 0). Individual plots can be detached for closer inspection and marked for different criteria. While multiple arrays are currently mapped into one window, the possibility to display multiple arrays in separate windows is under development.

The MANTA GUI aims to be flexible and dynamic, while occupying minimal screen space and providing context (mouse-over) help, to reduce the need for accessing help files during normal operation. The GUI consists of the control window (Figure 2A), several smaller settings windows [recording channel selection (Figure 2B), across-channel referencing (Figure 2C) and audio channel selection (Figure 2D)] and the plot window (Figure 3), which can be summoned when required.

The main control window (Figure 2A) allows the experimenter to select/set all options required for an acquisition session. The changeable options pertain to properties of the acquisition, the saved data, the displayed data and the audio output. All options from the configuration window can be saved and loaded in a future session from the GUI. Additional small panels allow to select recording channels per DAQ card (Figure 2B), choosing sets of channels to form a common reference for each other (Figure 2C, to counter common noise), and selecting channels for audio output (Figure 2D).

The display window contains one plot for each channel in a location corresponding to the geometry of the recording probe. Depending on configuration, multiple plots can be displayed for each channel, such as differently filtered versions of the signal, the spike threshold, and the spike rate. If triggered spikes or the short-term Fourier spectrum are selected, the display dynamically changes to add this information. Individual channels can be transferred to separate windows for more detailed display and inspection. The magnification of the data can be controlled with the mouse-wheel or by clicking inside the window. Generally, hovering the cursor above an element of the GUI, displays some additional information for the specific element.

### Controlling acquisition

Two modes of acquisition are supported, locally controlled and remotely triggered. The locally controlled mode uses the buttons and fields in the user interface to start and stop acquisition, display and saving. This mode serves to prepare a recording or collect exploratory data. Acquisitions of precise length are not supported in this mode, since to this end, MANTA will typically be controlled remotely or triggers from the stimulus software will be saved in some analog channels for later synchronization.

The remotely triggered mode is used for temporal synchronization and automated collection of many trials. The remotely controlled operation consists of two stages for preparing and starting a recording. First, a TCP/IP communication prepares the recording, i.e., defining internal structures and setting up the files required for saving the data (see below for details). Second, a digital (0–5 V) signal connected to a PFI/DIO line on one of the analog/digital DAQ cards is used to actually start acquisition. Trials can have arbitrary length, which allows flexible acquisition, such as when the trial length is determined by the behavior of the animal. Acquisition is terminated by another TCP/IP communication.

The TCP/IP communication is based on a standard TCP/IP connection, which can be established using a general TCP/IP client. MANTA is acting as a TCP/IP client in this communication, which will allow multiple MANTA clients to be connected to one controlling server for larger acquisition tasks.

The TCP/IP connection only has to be established once after starting MANTA, after which point all DAQ control can be transferred to the controlling program/stimulator. The TCP/IP communication follows a simple, extensible syntax, which at this point provides commands the standard operations during data-acquisition (see Table 2 for details). The syntax is **COMMAND** | **DATA !**, i.e., command and data are separated by a vertical bar and the message is terminated by an exclamation mark, e.g., **INIT** | **C:\ Data\ Animal\ Day\ File !** for initiating a recording or **SETVAR** | **foo = 1; !** for setting a variable on the receiver side.

**Table 2:** **TCP/IP commands**.

**Command**	**Description**	**Arguments**
INIT	Initialize a recording	Base filename to write to
START	Prepare for trial start (but wait for trigger)	Current filename (parsed in MANTA)
STOP	Stop a trial	–
COMTEST	Test communication	–
RUNFUN	Execute a Matlab function in the MANTA workspace	String of the function to execute
GETVAR	Transfer variable from MANTA to the controller (as a string)	Name of the variable
SETVAR	Transfer variable from the controller to MANTA (as a string)	Name of the variable

TCP/IP communication only adds a delay of a few milli-seconds between trials and thus does not slow down acquisition noticeably. Intertrial delays are on the order of a few 100 ms, in which data files are closed and opened, and runtime information is written to disk. If required this time can be shortened further by reducing repetitive tasks (e.g., avoid reinitialization of channels, increase loop speed to detect stop signal faster).

### Performance

Prompt audio-visual feedback in a DAQ system permits the user to observe relationships between evoked activity and externally-controlled stimuli. Conventionally, graphics display in MATLAB is a performance bottleneck, preventing real-time applications which require substantial data display. To improve the speed of the visual display we make use of MATLAB's ability to update an existing plot via references to the graphics objects rather than fully redrawing them. To further optimize the visual display, the number of data points is matched to the size of the plot windows on the screen. These techniques lead to a substantial gain (>20X) in display speed and allow us to draw 128 channels in ~75 ms. Depending on how much processing (filtering, triggering, threshold-computing, averaging) the user chooses to perform at a given time, the exact processing speed will vary.

**Figure 4:**
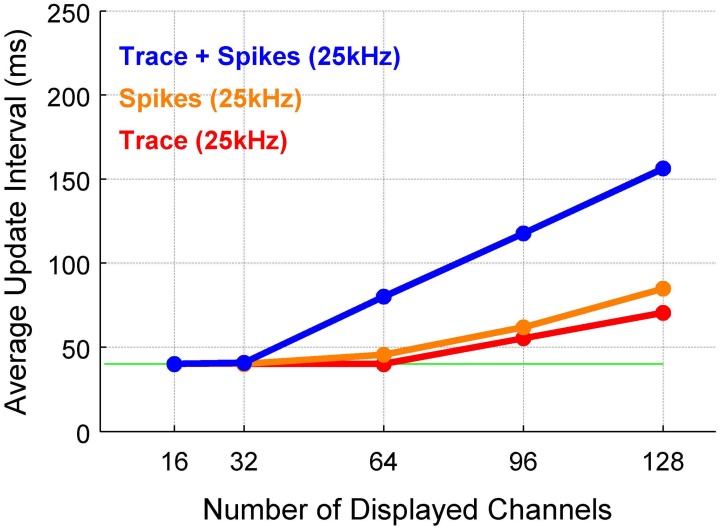
**The update interval remains usable up to a high number of channels.** The time between successive updates of the display depends on computer hardware, the analyses displayed and the parameters of the acquisition. The dependence of update rate on the number of channels is close to linear, only flattened at the lower limit by the maximum frame rate (25 Hz = 40 ms). The update interval is strongly influenced by whether only the raw trace (red), only spikes (red), or both are displayed (blue). The dependence on the sampling rate was only slight, since only a fraction of the points are displayed in each step (data not shown).

Average update intervals for plotting the voltage trace, adding spikes and adding automatic threshold computation are shown in Figure 4. If only the traces (red) or spikes (orange) are displayed, the update speed exceeds 10 Hz for 128 channels. If both are displayed together the performance reduces to ~6.5 Hz. Results for analog and digital headstages are not distinguished, since extracting the data from the DAQ cards constitutes only a few percent of the overall processing time and thus leads to very similar overall values.

The present performance results were collected on a Windows 7 (64-bit) system. Graphics performance, especially 2D performance, can be substantially faster on Linux systems, which can amount to 30% on the same hardware. However, graphics performance is substantially slower on Mac systems, owing to the suboptimal X11 implementation/integration.

MATLAB graphics performance depends mainly on the processor speed, rather than on the speed of the graphics card. With new processors adding more cores and increasing the clock-rate, the display performance of MANTA will increase as well, as many core algorithms in MATLAB are multithreaded. Thus, the results could be improved if the latest generation of processors were used.

Displaying the voltage traces of many hundreds of channels will be close to impossible at the moment, both with respect to display space, as well as update rates in MATLAB (or human perception). However, the Mathworks is currently overhauling MATLAB's graphics abilities to support specialized graphics hardware. Alternatively, a more efficient visual representation of such large datasets may be in order, e.g., 2D heatmaps/3D vector maps of instantaneous rate, which can be displayed rapidly (>30 Hz for 1000 × 1000 map) in MATLAB.

### Reliability

**Figure 5:**
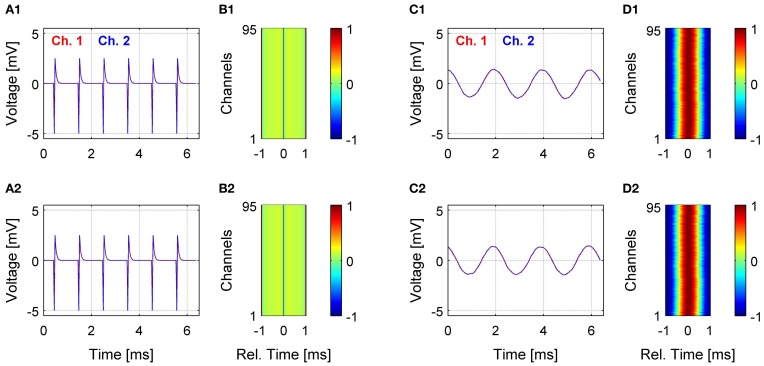
**Reliability of data acquisition over extended recording times.** The reliability of the system to acquire samples with precise timing was tested on two levels, only including the digital DAQ card **(A,B)** and with an electrode and the digital headstage connected to the digital card **(C,D)**. In the former case, a repeating pattern was created digitally, in the latter case, a 500 Hz sinusoudal voltage signal was injected into the bath with the electrode. All recordings were performed at 31.25 kHz on 96 channels for 1 h of recording time. The top row shows results after a few seconds of acquisition, and the bottom row shows the same channels after an hour of recording. As expected, the channels superimpose closely at both times **(A,C)**, which is also reflected by the correlation structure between all channels **(B,D)**. In the case of the digital DAQ card, only a single sharp peak in correlation exists at 0, whereas for the condition with the sinusoidal signal, the correlation exhibits the expected sinusoidal shape. The residual jitter in the peak of correlation in the latter case is attributable to the filtering properties of the electrode. The signal amplitudes in the latter case were equalized to correct for differences in impedance between different electrodes.

A central requirement of a DAQ system is to acquire data from multiple channels without dropping samples and maintaining precise synchronization across channels. Acquisition in MANTA is based on the proven DAQ drivers by National Instruments, HSDIO for digital acquisition and DAQmx for analog acquisition. While these drivers are unlikely to drop or misalign samples, interaction between MANTA and the driver, particularly under conditions of high computer load, could entail problems of this kind. We tested the reliability of MANTA to acquire samples without error in two configurations for the digital system (Figure 5). First, we created a periodic, digital signal, simulating the output of the digital headstage and recorded it with the digital DAQ card for 1 h at 31.25 kHz (Figures 5A/B). To check whether any samples were missed during this period, we computed the peak-to-peak difference for the periodic signal, which was constant for the entire period (data not shown). Also, the channels stayed perfectly in sync from the beginning (Figure 5A1) to the end (Figure 5A2) of the recording (see Figures 5B1/2 for the correlation of all channels with channel 1). Second, we connected a 96 channel electrode to the digital headstage and injected a 500 Hz sinusoidal voltage signal into the electrode bath, again for 1 h at 31.25 kHz (Figures 5C/D). Similarly, the channels stayed in sync over the entire period (Figures 5C1 vs. 5C2, note, that the amplitudes were equalized between the channels to account for different electrode impedances). The correlation between the channels in this case resembles the shape and period of the 500 Hz sinusoid (Figures 5D1/2).

Regarding asymptotic reliability, we have not directly tested whether a regular PC can sustain the acquisition of the theoretical maximum of 1440 channels at 31.25 kHz. Acquisition at this rate would amount to ~90 MB/s (at 16-bit resolution), which should be well within reach of currently available hardware, given that the data transfer rates exceed the required amount by more than a factor of 5 (DDR3 RAM: >12 GB/s, PCIe bus: >500 MB/s, SSD harddrive: >500 MB/s). Similarly, we estimate the processing required for 1440 channels to be around 60% on a current multicore processor (e.g., Core i7, 3770 K). For the analog front-end, the maximal required data rates are smaller (~30 MB/s for 576 channels), and this system is therefore not expected to pose any additional problems.

### Usability

A recording system should not only be stable and accurate but also easy to use and control, given the wide range of expertise of potential users in an interdisciplinary field. To assess whether MANTA fulfills this criterion, we asked the current set of MANTA users (*n* = 7) a set of questions regarding usability and user level performance in a questionnaire (which could be returned anonymously). The individual questions and the corresponding responses are summarized in Table 3. Overall MANTA was rated highly with respect to performance and usability, also in comparison with other DAQ systems. We also asked about missing features, which suggested online spike-sorting as a desirable feature, and has since been added. The online repository of MANTA will permit users to submit bugs and feature requests using a standard tracking system.

**Table 3:** **Usability and performance questionnaire (*n* = 7)**.

**Question**	**Possible answers**	**Responses (ø ± *SD*)**
Is the performance (graphics, recording, etc.) responsive?	[−2,−1,0,1,2]	1.64 ± 0.48
How long do you estimate it takes to learn to use MANTA?	Number in minutes	36 ± 41
How would you rate the usability of MANTA?	[−2,−1,0,1,2]	1.36 ± 0.48
Are the features offered by MANTA less or more than in other DAQ systems?	[−2,−1,0,1,2]	1.29 ± 0.76
Is the handling of MANTA better or worse than with other DAQ systems you have worked with?	[−2,−1,0,1,2]	1.57 ± 0.53

### System costs

**Figure 6:**
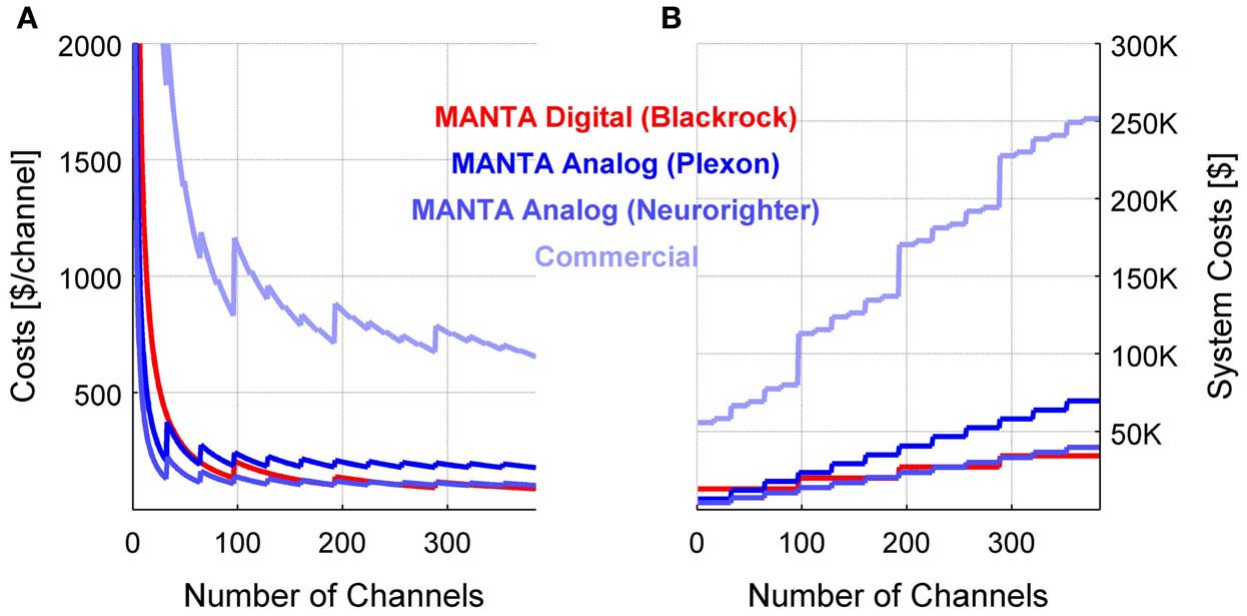
**Comparison of costs per channel or system costs for analog and digital systems.** Acquiring neurophysiological data from large electrode arrays necessitates matched numbers of channels in the DAQ system. A MANTA based DAQ system can be built with hundreds of channels at moderate costs, since per channel costs are <$100/channel **(A)** and system costs even for a 400 channel digital system (red) can stay below $50 K **(B)**. The MANTA/Neurorighter system stays similarly affordable (medium blue). The MANTA/Plexon system is about 50% more expensive, but has the advantage of not requiring any custom hardware components. Commercial solutions usually incur significantly higher costs. To avoid singling out one of the commercial systems, we report here the average and the distribution for a range of systems from various vendors.

For commercial systems, dedicated hardware and per-channel software licensing fees are the main drivers for high costs of many-channel systems. The use of off-the-shelf hardware in combination with open-source software allows to significantly reduce the price of such a system (Figure 6). We provide the per channel costs (Figure 6A) as well as entire system costs (Figure 6B), for systems with up to 400 channels. Since the per-channel costs almost reach their asymptote at this point, larger systems can be easily extrapolated. In comparison with commercial systems, we compute an average price over multiple vendors (Plexon, Tucker Davis Technologies, Alpha Omega, Blackrock), to avoid promoting or disparaging a single vendor. The costs listed here contain the entire setup, including the licensing costs, DAQ-hardware, headstages, cabling, and a computer with a large screen. Costs for MATLAB and toolbox licenses were not included in the system costs here, since they are usually available to labs through a university multi-seat license. Including them would add ~$1600 for a given setup, independent of the channel numbers, and thus decrease on a per-channel basis for large scale systems.

### Setup time

The setup time for the three MANTA system variants presented here depends on the number of channels and the background of the scientist. The MANTA/Plexon combination is the fastest, requiring essentially only the setup of the computer and connecting the output of the amplifier to the break-out boxes (which requires only a simple ribbon cable with a 0.1″ pitch, dual row, 34-pin connector). Per 16 channels/break-out box, this consumes about 1–2 h. Hence, altogether a system with a few hundred channels can ideally be setup in ~2 days.

The other two systems require the additional step of ordering a PCB (files are provided in the Supplementary Materials), acquiring electronic components (parts list provided online) and soldering the components onto the PCB. Depending on the level of soldering experience, this can be achieved in ~3 h/16 channels for the Neurorighter system and in ~2 h/96 channels for the digital Blackrock headstage. Hence, the entire setup time for the Neurorighter system is ~1 week, and a few days in the case of the digital Blackrock system.

### Sample recordings

**Figure 7:**
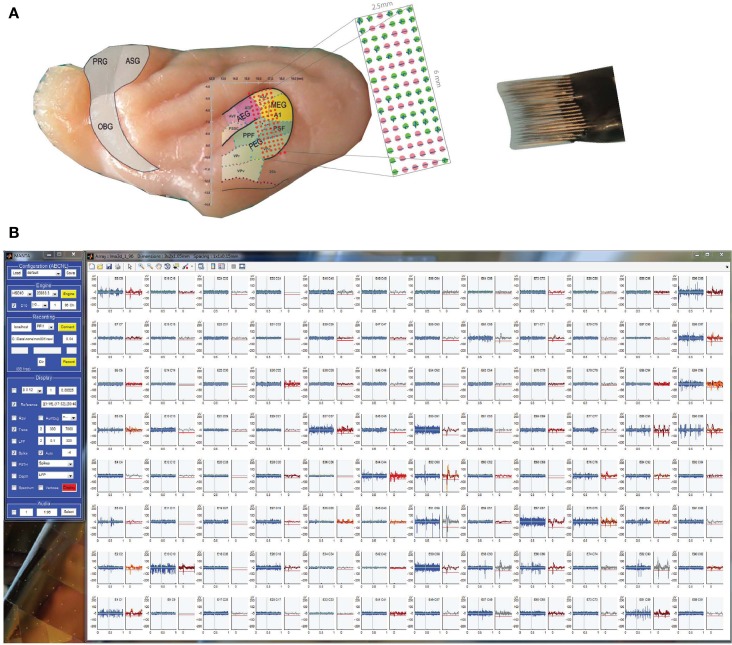
**Example recordings from a 96 channel array in auditory cortex of the ferret. (A)** A rectangular micro-electrode array (right) was inserted at a range of depth in the auditory cortex, covering a range of subareas simultaneously, among them primary (dorsal, A1, AAF, yellow) and secondary (ventral, PEG, PPF, green) areas. The detailed locations are indicated by the channel map superimposed on a generic ferret brain (brain preparation and image courtesy of Dr. Susanne Radtke-Schuller). Locations with spikes at this depth are indicated by a green circle in the magnified representation. **(B)** Raw data traces for each channel in the array during a pure tone tuning stimulus, with both voltage trace and spike display active.

In the first example, we recorded from the primary auditory cortex of an awake ferret using a 96 channel microelectrode array. The geometry of the array was planar, i.e., a two dimensional grid of electrodes in a 16 × 6 layout, spanning both primary and secondary areas in the auditory cortex (Figure 7A). The arrangement in Figure 7B is configured to represent the physical layout (when looking from the back of the probe) thus providing a natural alignment. A total of 42 channels showed spikes in this particular recording, which are shown to the left of each recording channel, and amounted to 56 neurons after spike sorting.

**Figure 8:**
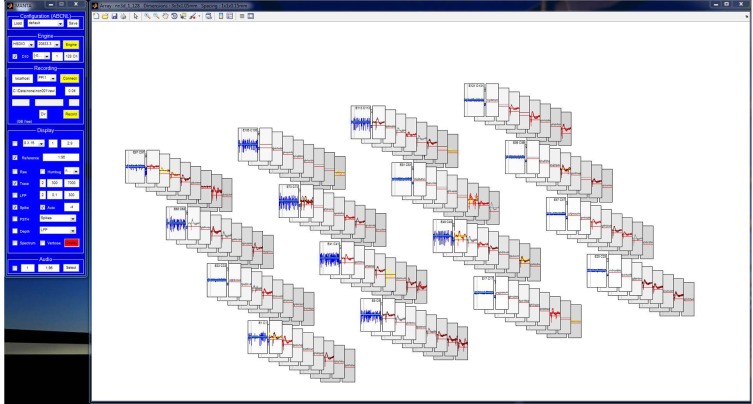
**Simulated recordings from a 128 channel (8 × 4 × 4), 3D array illustrating the ability to accurately visualize array geometry.** The location of the axes corresponds to the physical location of the electrodes. Dragging the plot rotates the array smoothly in 3D, and the background color of the axes indicates the distance of plot from the view plane. This plot also illustrates MANTA's simulation mode, which allows for offline development, i.e., spikes, line noise, slow noise, and background noise are simulated for testing filtering and display functionality.

In the second example, we illustrate the capability to visualize the geometry of the recording probe (Figure 8). Aside from the possibility to define arbitrary two dimensional locations of electrodes (and their associations with internal channels), MANTA allows the visualization of 3D probe geomtries. We chose a geometry that mimics one of the 128 channel 3D probes currently available from NeuroNexus. The plots show data simulated by MANTA's simulation mode, which generates a random background with a random number of realistically shaped “neurons” (spike-shapes) per channel embedded (for testing spike-sorting).

The 3D view mode is internally detected from the probe geometry if electrode locations have been provided in a configuration file. The physical locations of the electrodes are used to generate logical locations of the corresponding plots. The assembly of plots can be rotated by dragging with the mouse outside the individual axes. The distance of the individual electrodes from the viewing plane is indicated by the darkness of the background color of each plot.

## Discussion

Open-Source DAQ systems can provide an important tool for neural systems analysis by reducing costs and thus enabling larger-scale experiments. In addition to minimizing experimental costs per channel, MANTA represents an attempt at a system that can be used by a wide range of users. MANTA aims to take a middle ground between extensibility and performance, achieving fast updating for high channel-counts and allowing for a maximum of flexibility in adapting and extending the code.

### Comparison with other packages

The Neurorighter project (Rolston et al., 2009; Newman et al., 2013) is another open-source DAQ system based on C#, targeting similar usage scenarios, i.e., multichannel recordings. While the update rate of the display is expected to be higher in Neurorighter, extensibility is more involved due to the choice of a compiled, rather than an interpreted language. Many changes in MATLAB can be implemented, tested and improved even while acquisition is running, and thus allow for a rapid iteration cycle for code development.

The Ephus system (Suter et al., 2010) is another project based on MATLAB which is targeting *in-vivo/in-vitro*, few channel recordings integrated with optical methods and stimulation. While in principle suitable for array recordings, Ephus is not optimized for this purpose, and would thus require substantial modifications to serve the same purpose as MANTA.

Hence, MANTA falls in a different usage scenario than other available software suites, focusing on array recordings with high number of channels from analog and digital headstages, but retaining the fast development cycle enabled by MATLAB.

### Comparison with commercial products

The decision whether to use a commercial or an open-source recording system is not trivial, as it depends on multiple factors. While we have emphasized throughout the increased flexibility, dynamics and cost efficiency of an open-source system, commercial systems provide other advantages. One advantage is the availability of support and maintenance by the vendor. Another advantage is the focus on a stable product. Ideally these two factors can be served by an open-source system as well, however, only if the development is split into a stable and an unstable version. The unstable version is the current working version of the developers, which brings the richest set of features along, but is less well tested, whereas the stable version is only amended by bug fixes. We have adapted this development model using the modern version control system git, which supports different development branches to coexist and flexibly merge them if needed.

### Choice of the MATLAB environment

The use of MATLAB requires users to acquire at least one single machine license, and limits modifiability at a certain level (e.g., the user has little control over the low-level graphics routines). Python is an increasingly popular open-source programming language that shares several features of MATLAB, especially with respect to fast development cycles. Opinions on the relative performance in terms of data processing and graphics display diverge, although there are exciting and high-performing graphics packages under development (e.g., Galry, http://rossant.github.com/galry/).

The reasons for choosing MATLAB as a basis was a mixture of various factors, mostly the authors' previous experience with MATLAB, availability of a rich set of GUI interactions and the current prevalence of MATLAB programmers in the neuroscience community. A Python based “*PYNTA”* reimplementation with potential performance benefits would be an interesting enterprise, although it is not entirely clear how easily the entire functionality on the GUI level could be ported to the new platform.

While MATLAB is generally available to most labs at no or low cost via a university-wide license, it would be worthwhile to reduce MANTA dependence on additional toolboxes. Currently, MANTA makes use of three MATLAB toolboxes (Signal Processing, Instrument Control, DAQ). The use of the Instrument Control toolbox could be avoided if an open-source TCP/IP server/client solution would be used (e.g., jTCP, which, however, does not support message triggered execution of functions). The DAQ toolbox is only used for audio output, which could be avoided if the data could be relayed via a third-party audio program. The Signal Processing Toolbox could be replaced by reprogramming some filtering operations directly, which could, however, be complicated by MATLAB's effective use of multi-threading, often more efficient than traditional, single threaded C/MEX code.

### Future developments

We are in the process of expanding the scope of MANTA in several directions, notably for supporting *multiple arrays* (e.g., for studies of multiple brain areas, neural data will be obtained from multiple arrays with different geometries, which should be treated separately in the display), *multiple computers* (with more than 256 channels, display space/speed will be a major hurdle, which could be alleviated by spreading acquisition over multiple computers), *adaptive stimulation control* [to support flexible, activity based stimulation, probably in combination with the stimulation hardware by the Neurorighter system by Rolston et al. (2009), Newman et al. (2013)], *NeuroNexus array geometries* (allows selecting an array using NeuroNexus syntax/from the NeuroNexus catalog), *advanced filtering/denoising* [for multielectrode recordings advanced filtering techniques can be used to improve signal quality, e.g., Dynamic Source Separation (DSS, de Cheveigné and Simon, 2007, 2008a,b)] and *alternative data displays* (e.g., 2D heatmaps/3D vector maps of instantaneous rate).

### Conflict of interest statement

Mike Sorenson is an employee of Blackrock Microsystems, which produce headstages that can be used with the MANTA system. He provided information on how to interact with the Digital Headstages at high speeds. The other authors declare that the research was conducted in the absence of any commercial or financial relationships that could be construed as a potential conflict of interest.

## Supplementary material

Video 1 **Dynamic interaction with MANTA during recording**.
